# Research progress in photochemical synthesis of urea through C–N coupling reactions

**DOI:** 10.1039/d5ra08170j

**Published:** 2025-12-09

**Authors:** Zhidong Yang, Peixia Li

**Affiliations:** a School of Chemistry and Environment, Ankang University Ankang 725000 Shanxi China yangzd1618@163.com

## Abstract

Urea, as an important organic compound, plays significant role in promoting the development of agriculture, industry and biological sciences. Conventional urea synthesis process requires harsh reaction conditions (high temperature and pressure), making it energy-intensive, emission-intensive, and costly. Photocatalytic C–N coupling is a potentially green and promising alternative strategy for synthesizing valuable urea from CO_2_ and inexpensive nitrogen sources (such as N_2_, nitrates, ammonia) as feedstocks under ambient conditions using solar energy. However, the specific details of urea photosynthesis have not been systematically reviewed so far. This article reviews the basic principles of photocatalytic urea synthesis, including the fundamentals, key intermediates, product identification and quantification. Meanwhile, it comprehensively summarizes research advances in photocatalytic urea synthesis, with a focus on the application and design principles of photocatalysts in urea production. Finally, the major challenges and prospective research directions in the field of photocatalytic urea synthesis are thoroughly discussed. We hope that this review will provide useful insights to inspire future research and discoveries.

## Abstract 2

Reserach advances in photocatalytic urea synthesis highlight fundamentals, innovative photocatalysts, mechanisms, and efficiency breakthroughs, paving significant guidance for sustainable energy conversion and energy-saving fertilizer production.

## Graphical abstract

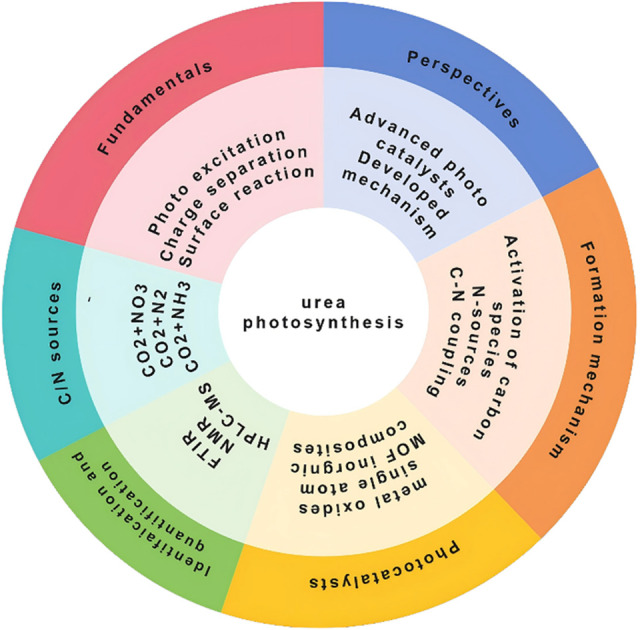

## 1 Introduction

Urea is a compound with critical applications across multiple fields.^1,2^ In agriculture, it serves as an efficient nitrogen fertilizer that improves soil quality, promotes plant growth, and enhances crop yield and quality.^3,4^ In industry, it is an important raw material for fine chemicals such as plastics, resins, coatings, adhesives, pigments, and dyes.^5,6^ In the energy sector, urea can be used as a proton conductor in fuel cells to improve performance, and it can also produce hydrogen through pyrolysis or electrolysis, serving as a potential hydrogen source.^5^ In medicine, urea exhibits functions such as keratin softening, wound healing, antipruritic, and antibacterial effects, making it widely used in dermatological treatments.^7,8^ Therefore, the development of the urea synthesis industry is of great significance for ensuring global food security, advancing chemical technology, promoting economic growth, and improving human living standards.^9^

The industrial urea synthesis process involves the reaction of ammonia and carbon dioxide under harsh conditions to produce ammonium carbamate, which is then dehydrated to produce urea.^10–13^ As one of the reaction feedstocks for urea synthesis, approximately 80% of the ammonia is derived from the Haber–Bosch process.^14^ The Haber–Bosch process requires high-temperature and high-pressure conditions (400–650 °C and 200–400 bar), accounting for 1–2% of global energy consumption.^14–16^ Hydrogen, as another key reactant in the Haber–Bosch process, is typically produced *via* steam methane reforming (CH_4_ + 2H_2_O → 4H_2_ + CO_2_) or coal steam reaction (C + 2H_2_O → 2H_2_ + CO_2_), both of which generate significant CO_2_ emissions.^17^ It is estimated that the Haber–Bosch process contributes to about 1.5% of global anthropogenic CO_2_ emissions.^17^ As a result, conventional urea synthesis not only consumes substantial energy but also exacerbates environmental burdens. It is urgent to explore more environmentally friendly and energy-saving urea production processes and technologies to reduce environmental pollution and resource consumption.^18^

Fortunately, solar-driven photosynthesis techniques are able to initiate chemical reactions under mild conditions, thereby reducing energy consumption and environmental issue and offering prospects for sustainable development and green chemistry.^19^ Photocatalytic C–N coupling reactions can convert carbon species such as CO_2_, CO, methanol and nitrogen species such as N_2_, nitrite, nitrate, or NO into valuable urea under ambient conditions and in aqueous solutions.^20–23^ Photosynthesis of urea not only makes use of abundant and free solar energy to alleviate the excessive use of fossil fuels and save energy, but also facilitates efficient and economical CO_2_ fixation, which is of great significance to solve the problems of resource scarcity, environmental issues, global warming. In particular, directly coupling N_2_ and CO_2_ to synthesize urea by photocatalysis have attracted much attention in recent years.^24–26^ In the process of photocatalytic urea synthesis, excited semiconductors first generate electrons and holes that participate in reduction–oxidation reactions to form nitrogen and carbon-containing intermediates. This is followed by the critical step of C–N coupling, and finally, electron-driven hydrogenation through proton-coupled reduction ultimately yields urea. Photocatalytic synthesis of urea is a prospective and innovative green chemical method, which opens up a sustainable pathway for urea synthesis.

**Fig. 1:**
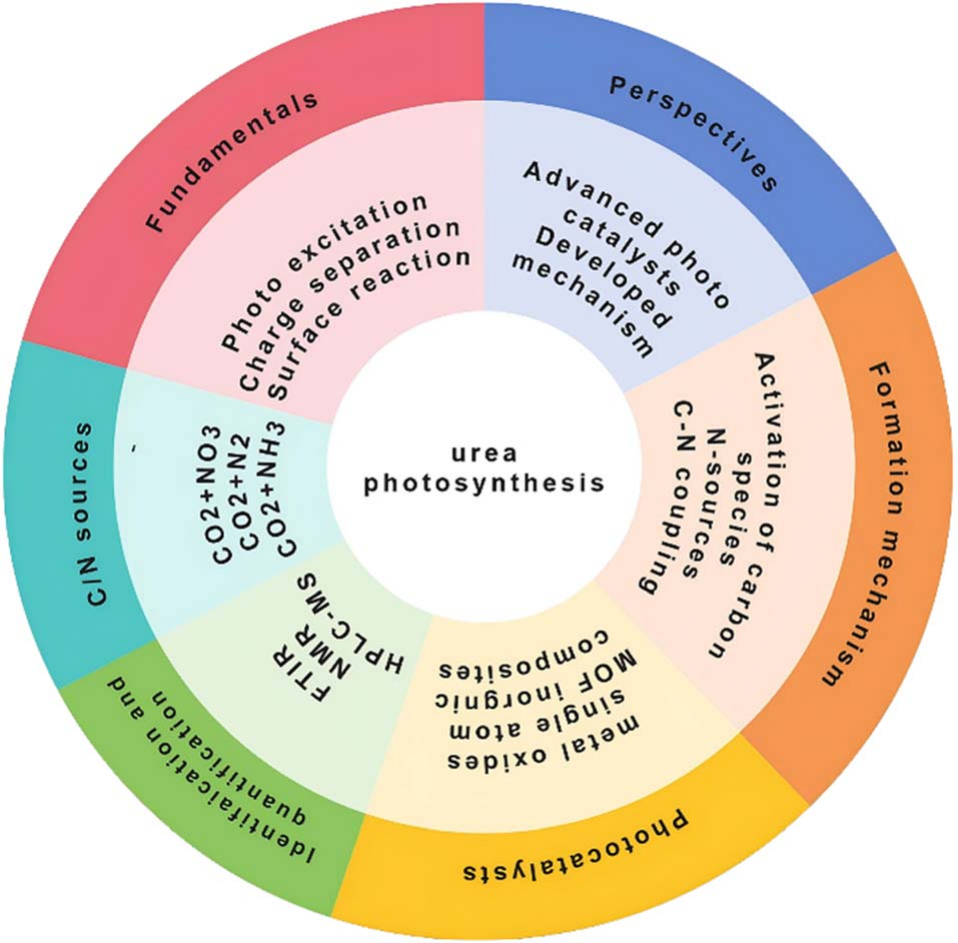
Core framework of this perspective for urea photosynthesis.

Although photocatalytic synthesis of urea has been intensively investigated and made some progress in recent years, reported photocatalytic urea synthesis rates remain unsatisfactory. Currently, there are several challenges for the photosynthesis of valuable urea, such as high thermodynamic stability of reaction substrates (*e.g.*, nitrogen and carbon dioxide),^27–30^ undesirable adsorption capacity of substrates/intermediates/products on the surface of the photocatalysts,^31^ high charge-carrier recombination efficiencies,^32^ slow kinetic processes of multi-electron transfers,^33,34^ and competing reactions in parallel products.^35,36^ These factors collectively result in low solar-to-chemical energy conversion efficiency, severely hindering the development of photocatalytic urea synthesis. In order to improve the photosynthesis performance of urea, a comprehensive understanding is essential to enable rational design of photocatalysts and systematic optimization of the photochemical reaction system. Herein, we present a systematic review of research advances in photocatalytic urea synthesis. The fundamental principles of the photocatalytic urea formation process are first introduced, covering the detection methods of key intermediates, as well as experimental identification and quantification techniques for urea and other parallel products. Subsequently, the performance of both classical and emerging photocatalysts across various reaction systems is summarized, with emphasis on their structure–activity relationships. Finally, current challenges in this field are discussed, and future research directions are outlined (Fig. 1). It is hoped that this review could provide an overview of the current research achievements related to the photocatalytic urea synthesis, thus stimulating the research on the design and synthesis of photocatalysts and the development of more efficient and stable photocatalytic systems.

## 2 The basic principle details for photocatalytic urea synthesis

### 2.1. Fundamentals of photocatalytic urea synthesis

Photocatalytic urea synthesis is a green synthetic method that utilizes renewable solar power to convert nitrogen-containing small molecules (*e.g.*, N_2_, NO_3_^−^, or NH_3_) and carbon sources (*e.g.*, CO_2_, CO or CH_3_OH) into urea.^37–40^ Its basic principles involve multiple complex steps, mainly including: (1) the absorption of light energy by the photocatalyst, (2) the generation and separation of photogenerated carriers, (3) the adsorption and activation of reactants, and (4) the formation of the urea by C–N coupling reactions.

**Fig. 2:**
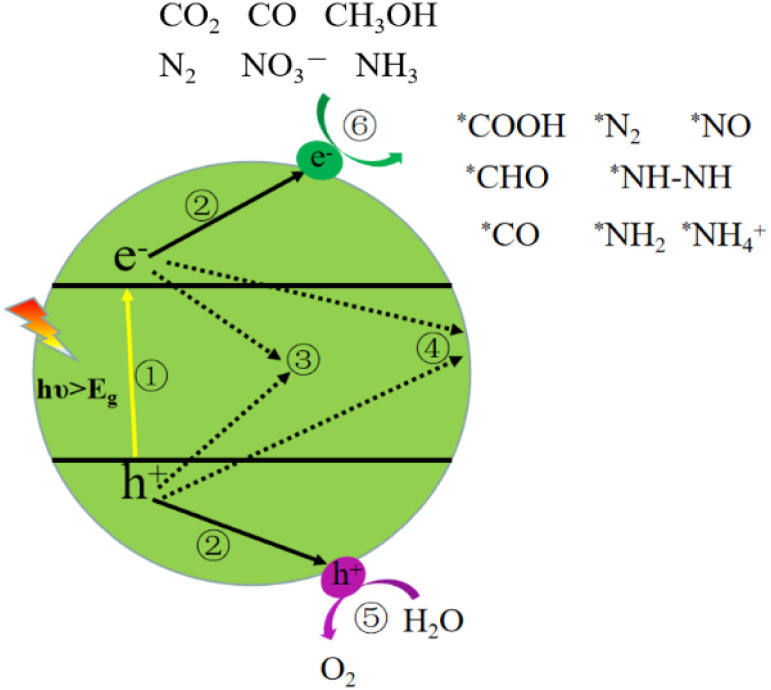
Schematic diagram of photocatalytic urea production.

The main stages in photocatalytic urea synthesis are shown in Fig. 2. When a photocatalyst is exposed to light with an energy greater than its bandgap width, electrons in the valence band are excited and transition to the conduction band, forming photogenerated electron (e^−^)–hole (h^+^) pairs. Then, the separation and transfer of photogenerated electrons and holes. There are several situations regarding the separation and transfer of photogenerated carriers. One is that the photoexcited electrons and holes are transferred to the reduction and oxidation sites on the semiconductor surface, where they undergo reduction and oxidation reactions with the adsorbed substrate molecules respectively, as in process ⑤, ⑥; another is that the excited state carriers undergo recombination during the process of separation and transfer, that is, bulk phase recombination, as in process ③; the third is the electron–hole recombination that occurs on the semiconductor surface, namely surface recombination, such as process ④. It is worth noting that the recombination of carriers severely hinders the solar energy conversion efficiency. Therefore, neither bulk phase nor surface recombination is conducive to the progress of photocatalytic reactions. Furthermore, adsorption and activation of reactants: carbon species and nitrogen sources are adsorbed on the surface of the photocatalyst and activated by photogenerated carriers. Finally, the activated intermediates undergo a C–N coupling reaction to form urea precursors which then proceed a series of hydrogenation and electron transfer processes to ultimately produce urea.

#### 2.1.1. Activation of carbon species

In the photocatalytic urea synthesis reaction, the reduction of CO_2_ is a critical step in forming the carbon source of urea. It is generally believed that CO_2_ is to form *CO intermediate, which then reacts with nitrogen-containing intermediates to generate urea. The specific analysis is as follows: the adsorbed CO_2_ molecule couple with one proton and one electron to form the adsorbed *HCOO (eqn (1)) which then further are reduced into *CO intermediate by the proton-assisted single electron transfer process (eqn (2)). The generated *CO intermediates couple with nitrogen-containing intermediates (such as activated N_2_, *NH_2_, *NO *etc.*) to form a C–N bond, ultimately generating urea. For CH_3_OH as the carbon source system, more·CH_2_OH radicals are generated from CH_3_OH, which are subsequently oxidized to *CHO intermediates on Pt cluster/TiO_2_.^40^

1 *CO_2_ + H^+^ + e^−^ → *COOH

2 *COOH + H^+^ + e^−^ → *CO + H_2_O

#### 2.1.2. Activation of N-containing sources

The N_2_ molecule is the most stable known diatomic molecule, possessing a very short N≡N bond length of only 109.8 pm with a bond strength of 941 kJ mol^−1^.^41^ A large energy gap (10.82 eV) exists between the highest occupied molecular orbital (HOMO) and lowest unoccupied molecular orbital (LUMO) of N_2_, whilst the molecule has both a high ionization potential (15.85 eV) and a low electron affinity (−1.9 eV) making oxidation or reduction difficult.^42^ The efficient activation of N_2_ is a key step in urea synthesis. N_2_ activation proceeds *via* two principal pathways: reduction and oxidation–reduction. In reduction systems, such as Cu SA-TiO_2_ (ref. 43), Pt cluster/TiO_2_ (ref. 40) and Pd–CeO_2_ (ref. 44), N_2_ is adsorbed onto metallic sites like Cu, Pt and Pd through “σ-π*” interactions and subsequently hydrogenated to yield *N_2_H, *N_2_H_2_, *NH_2_ (eqn (3)–(5)). Reductive sites in photocatalysts like Ti^3+^-TiO_2_, CeO_2−*x*_ and Ru–O_4_Ti_1_ facilitate multi-electron transfer to directly crack the N≡N bond. For the path of oxidation–reduction, N_2_ is first oxidized to nitrogen oxides, which are then coupled with *CO and gradually hydrogenated for reduction (eqn (6)–(8)). System like Ni1CdS/WO_3_,^45^ the N_2_ was converted into NO species by *OH radicals generated from photogenerated holes over the WO_3_ component, meanwhile, the CO_2_ was transformed into *CO species over the Ni site by photogenerated electrons. The generated NO and *CO species were further coupled to form *OCNO intermediate, then gradually transformed into urea. Other systems, such as nitrate and nitrite, mainly underwent hydrogenation reaction (eqn (9)–(15)).

N_2_ reduction:

3 *N_2_ + H^+^ + e^−^ → *N_2_H

4 *N_2_H + H^+^ + e^−^ → *N_2_H_2_

5 *N_2_H + 2H^+^ + 2e^−^ → 2*NH_2_

N_2_ oxidation–reduction:

6 2H_2_O + 4h^+^ → 2OH + 2H ^+^

7 N_2_ + 2OH → 2NO + 2H ^+^

8 NO + 4H^+^ + 4e^−^ → *NH_2_ + H_2_O

NO_3_^−^ reduction:

9 *NO_3_ + H^+^ + e^−^ → *NO_2_OH

10 *NO_2_OH + H^+^ + e^−^ → *NO_2_ + H_2_O

11 *NO_2_ + H^+^ + e^−^ → *NOOH

12 *NOOH + H^+^ + e^−^ → *NO + H_2_O

13 *NO + 2H^+^ + 2e^−^ → *N + H_2_O

14 *N + H^+^ + e^−^ → *NH

15 *NH + H^+^ + e^−^ → *NH_2_

#### 2.1.3. C–N coupling

Typically, the urea synthesis from CO_2_ and nitrogen-containing species is regarded as a co-reduction process. In general, C–N proceeds *via* three principal pathways. (1) *NH_2_ and *CO couple to form the *NH_2_CO (eqn (16)), which is followed by coupling with excessive *NH_2_ to produce urea. (2) *NO couples with *CO forming * ONCONO (eqn (17)). Subsequent stepwise hydrogenation of these intermediates yields urea (eqn (18)). (3) Activated *N≡N couples with *CO to form *NCON (eqn (19)), which then undergoes hydrogenation (eqn (20)). Additionally, system like Pt Cluster/TiO_2_,^40^ the crucial step of C–N coupling was initiated by the reaction between *NH─NH and *CHO intermediate. It was observed that Pt clusters undergo “σ–π*” acceptor–donor interactions with N_2_, resulting in a reduction of the N_2_ activation barrier and subsequent activation into *NH–NH intermediates. Furthermore, under the promotion of N_2_ molecules and Pt clusters, more ·CH_2_OH radicals were generated from CH_3_OH, which were subsequently oxidized to *CHO intermediates. Further analysis with DFT calculation demonstrated that *NH–NH and *CHO were important precursors in C–N coupling reactions.

16 *NH_2_ + *CO → *NH_2_CO

17 *NO + *CO → *ONCONO

18 *ONCONO + 8H^+^ + 8e^−^ → (NH_2_)_2_CO + 2H_2_O

19 *N_2_ + *CO → *NCON

20 *NCON + 4H^+^ + 4e^−^ → (NH_2_)_2_CO

### 2.2. Identification of key intermediates

Various intermediates are formed on the surface of the photocatalyst during activation of CO_2_ and nitrogen source, such as *CO_2_, *COOH, *CO, *N_2_, *N_2_H_2_, *NH_2_, *NH, *NO_2_, *NO_2_OH, *NOOH, *NO, ect. Among them, the determination of C–N coupling intermediates such as *NH_2_CO, *OCNO and *NCONO are particularly significant, which ultimately leads to urea formation. Therefore, the determination of key intermediates in the photocatalytic synthesis of urea is of great scientific significance for revealing the reaction mechanism and understanding the catalytic process.

**Fig. 3:**
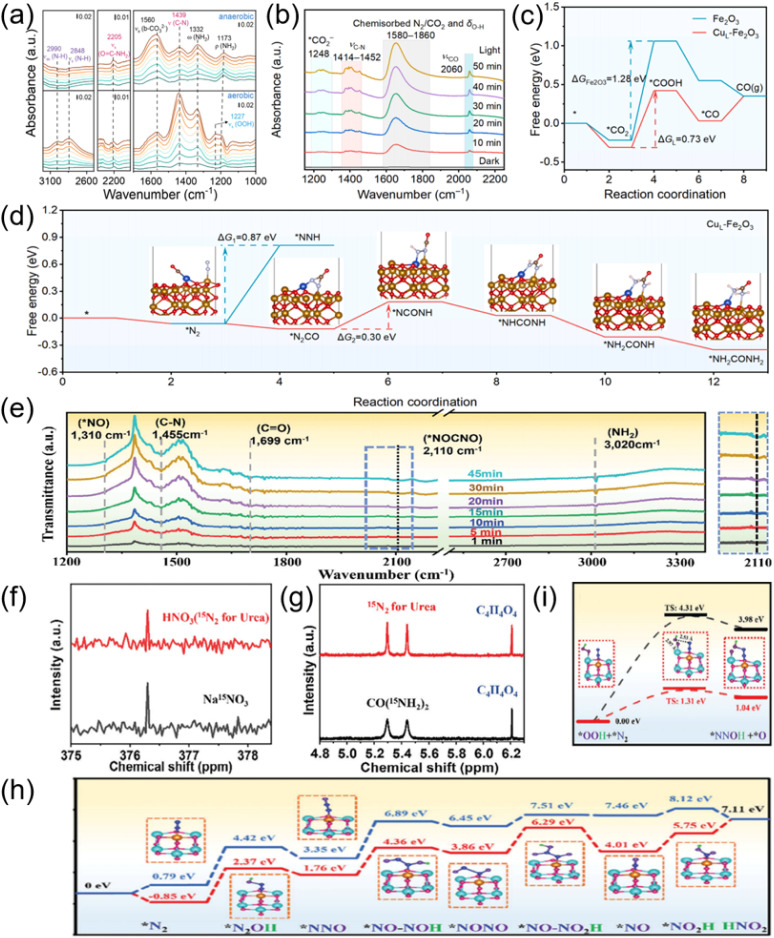
(a) *In situ* FT-IR spectra of the photocatalytic urea synthesis under anaerobic (80% CO and 20% Ar) or aerobic (80% CO and 20% CO) condition on P25-4 h. Reproduced with permission from ref. 46. Copyright 2025, Wiley-VCH. (b) *In situ* DRIFT of Ni_1_–CdS/WO_3_ during photocatalytic urea synthesis. Reproduced with permission from ref. 45. Copyright 2024, Wiley-VCH. (c) Gibbs free-energy profiles of the CO_2_ reduction process for Fe_2_O_3_ and CuL-Fe_2_O_3_. (d) Gibbs free-energy profiles and the geometric structure diagram of intermediates evolution during the urea synthesis process for CuL-Fe_2_O_3_ by alternating hydrogenation pathway. Reproduced with permission from ref. 37. Copyright 2025, Elsevier. (e) *In situ* ATR-FTIR spectroscopy measurements of Ru–TiO_2_ over time during the coupling of N_2_ and CO_2_. The enlargement at 2100 cm^−1^ is shown in blue on the right. (f) ^15^N-NMR spectra of ^15^NO_3_^−^ (standard) and test product from NOR using ^15^N_2_ as feeding gas. (g) ^1^H-NMR spectra of CO (^15^NH_2_)_2_ (standard) and test product from urea using ^15^N_2_ as feeding gas. (h) Calculated free energy diagram for possible NOR mechanism on TiO_2_ and Ru–TiO_2_ (blue line: TiO_2_; red line: Ru–TiO_2_). (i) Reaction barrier diagrams from *N_2_ to *NNOH on TiO_2_ and Ru–TiO_2_ (black line: TiO_2_; red line: Ru–TiO_2_). Reproduced with permission from ref. 48. Copyright 2024, Wiley-VCH.

Combining *in situ* characterization techniques with theoretical calculations can capture key intermediates, reveal active sites and reaction pathways, thereby deeply understanding the reaction mechanism and optimizing the catalytic system. *In situ* FTIR (DRIFTS) is an important technique for studying key intermediates in the photocatalytic synthesis of urea. It can monitor the dynamic evolution of adsorbed species on the catalyst surface in real time and reveal the reaction path. For example, Sheng *et al.* used *in situ* diffuse reflection infrared Fourier transform spectroscopy (*in situ* DRIFT) to monitor the photosynthesis process of urea. Upon illumination, the emergence and growth of several intermediate bands were observed in both cases (Fig. 3a). The two vibrational bands located at 1332 and 1173 cm^−1^ were attributed to the wagging and deformation modes of NH_2_ species, respectively, suggesting the one electron oxidation of NH_3_. For C–N intermediates, the 1439 cm^−1^ band corresponded to the C–N stretching vibration, closely resembling the standard band for urea, while the vibrational band located at 2205 cm^−1^ was assigned to the stretching mode of O=C–NH_2_, derived from the coupling of NH_2_ with CO.^46^ Zheng *et al.* utilized *in situ* DRIFT spectroscopy to track the evolution of the critical intermediates in reactions.^45^ As illustrated in Fig. 3b, an obvious broadening band at about 1594 cm^−1^ was observed, which could be attributed to the co-existence of the stretching mode of adsorbed NO species at 1590 cm^−1^ and the adsorbed CO species at 1610 cm^−1^. More importantly, they captured the *OCNO intermediates at 2120 cm^−1^, which was generated by C–N coupling from *CO and *NO. Theoretical calculations can predict the stability of intermediates, energy barriers and the reaction mechanism, complementing experimental data. By calculating the adsorption configurations and energies of possible intermediates (such as *CO, *NH_2_, *CONH_2_, ect), thermodynamically feasible paths were screened out. For instance, Pang *et al.* performed DFT calculations to explore the photocatalytic C–N coupling mechanism for urea production (Fig. 3c).^37^ Comparing the Gibbs free-energy (Δ*G*) profiles for CO_2_ reduction, revealed that pure Fe_2_O_3_ faced challenges in generating *CO because of its inherently high CO_2_ activation barrier (with a rate-determining step of 1.28 eV). In contrast, CuL-Fe_2_O_3_ exhibited a more moderate rate-determining step (0.73 eV), thereby facilitating the generation of *CO intermediates. In addition, the desorption barrier stabilized the *CO intermediates for subsequent C–N coupling reactions. Considering the molecular orbital matching between *CO and *N_2_, the formation of *N_2_CO intermediates was a spontaneous exothermic reaction (Fig. 3d). Coupled with mass spectrometry (MS) and nuclear magnetic resonance spectroscopy (NMR), isotope labeling enables precise tracking of reaction pathways. Zhang *et al.* detected *NCONO intermediate (2100 cm^−1^) on Ru–O_4_Ti_1_ sites using *In situ* ATR-FTIR spectroscopy coupled with ^15^N-NMR (Fig. 3e–g).^47^ DFT calculations revealed unique Ru nanostructure (Ru–O_4_Ti_1_), which effectively triggered the activation of inert N_2_ molecules, facilitated the formation of crucial *NN(OH) intermediates (Fig. 3h), lowered the energy barrier of the potential determining step (Fig. 3i), and served as an “electronic pump” for electron migration from nitrogen to TiO_2_ support during urea photosynthesis.

### 2.3. Product identification and quantification

Photocatalytic urea synthesis is accompanied with the generation of various by-products, including CO, CH_4_, C_2_H_4_, HCOOH, CH_3_OH, C_2_H_5_OH, H_2_, NH_3_, CH_3_NH_2_, C_2_H_5_NH_2_, CH_3_CONH_2_, *etc.* Gas chromatography can be used to determine gas-phase products. For liquid-phase or soluble products, the identification and quantification could be achieved by nuclear magnetic resonance (NMR) spectroscopy, the diacetyl monoxime method or the urease decomposition method, high-performance liquid chromatography (HLPC) and high-performance liquid chromatography-mass spectrometry (LC-MS) spectroscopy.

For the typical identification and quantification of urea by ^1^HNMR spectroscopy, dimethyl sulfoxide-*d*_6_ (DMSO-*d*_6_) (99.8 atom % *D* with 0.03% [v/v] trimethylsilane) and disodium maleate aqueous solution as internal standards are added into reaction solution without any preliminary treatment. Subsequently, the mixed solution is transferred to a NMR tube and analyzed. However, the signal intensity of active hydrogen will be influenced by pH value and solution concentration.

Regarding the diacetyl monoxime method, typically, 5 g of diacetylmonoxime (DAMO) and 100 mg of thiosemicarbazide (TSC) are dissolved in distilled water to prepare the DAMO-TSC solution with 1000 mL in total volume. Subsequently, the preparation of acid-ferric solution is mainly to add 100 mL concentrated phosphoric acid and 300 mL concentrated sulfuric acid to 600 mL deionized water, and then dissolve 100 mg FeCl_3_ in the above solution. For the quantification of urea, 1 mL of DAMO-TSC solution and 2 mL of acid-ferric solution were added to 1 mL of sample solution. After vigorous mixing, the mixed solution was heated to 100 °C and maintained at this temperature for 15 minutes. When the solution is cooled to 25 °C, UV-vis absorption spectra were collected at a wavelength of 525 nm. The concentration–absorbance curve is calibrated using a standard urea solution with different concentrations. This method requires strict control of reaction conditions (temperature, time), and its accuracy can be affected by nitrates, ammonia, *etc.*

Additionally, the quantification of urea is achieved by HPLC (*e.g.* Agilent 1220 Infinity II) spectroscopy. HPLC was performed on a Luna 5 µm NH_2_ column (250 mm × 4.6 mm). The corresponding mobile phase, flow rate, and detected wavelength is needed. Measurement of urea in the reaction product: firstly, the product was rinsed repeatedly with ultrapure water to ensure that it was adequately collected, then pumped and filtered and transferred to a volumetric flask and diluted to a volume of 50 mL. 5 mL of it was concentrated to 1 mL for the assay. The advantages of the HPLC method are high resolution, good repeatability, and less impurity interference. Compared with HLPC, LC-MS has higher sensitivity and is suitable for trace analysis, and reaction mechanism research in photocatalytic synthesis of urea, but the instrument and maintenance costs are relatively high.

Overall, it is necessary to balance the detection target (urea concentration, matrix complexity), equipment availability and data requirements when choosing specific method. For mechanism research, NMR (isotope tracking) or LC-MS (product identification) are preferred. The HPLC and DAMO-TSC methods are recommended for routine testing inclinical/environmental applications. For trace detection, the HPLC and LC-MS methods offer ultra-high sensitivity for detecting urea.

## 3 The progress of urea photosynthesis

### 3.1. Urea synthesis from CO_2_ and NO_3_^−^

In recent years, significant progress has been made in urea synthesis from inexpensive carbon and nitrogen sources. In particular, urea synthesis originates from CO_2_ and NO_3_^−^ from industrial emission or agricultural fertilizer.^4,15,48^

Photochemical synthesis of urea was first reported by Yoneyama in 1998.^49^ Inspired by the high activities for reduction of CO_2_ to methanol and the favourable reaction rate for the reduction of NO_3_^−^ to NH_4_^+^ of size-quantized TiO_2_ semiconductor nanocrystals immobilized in polyvinylpyrrolidinone gel film (Q-TiO_2_/PVPD), the group explored the simultaneous reduction of CO_2_ and NO_3_^−^ (from LiNO_3_) to form urea using Q-TiO_2_/PVPD in the propylene carbonate solution containing isopropanol as a hole scavenger. Apart from the target urea, the by-products formed included methanol, ammonia, hydrogen, Ti^3+^ and acetone. Except for acetone, which was the only observed oxidation product derived from the oxidation of isopropyl alcohol, the other five products are reduction products. The sum of the quantum efficiencies of the reduction products (15.4) was very close to that of the oxidation products (15.9). Besides, NH_2_OH and NO were also used as nitrogen sources instead of NO_3_^−^, and HCOOH and CO gas were used instead of CO_2_ as carbon sources. All cases tested in the study gave urea, but the reaction rate and distribution of products were largely influenced by the combination of carbon and nitrogen sources (CO_2_–NO_3_^−^, CO–NO_3_^−^, HCOOH–NO_3_^−^, CO_2_–NH_2_OH, and CO_2_–NO) used. It should be noted that the reduction of NO_3_^−^ was hypothesized as the rate-determining step in the urea photosynthesis reaction, as evidenced by the significant increase in urea yield when the nitrogen source is switched to NO or NH_2_OH, both of which are intermediates in the reduction reaction of NO_3_^−^.

Subsequently, they studied that photocatalytic reduction of CO_2_ in the presence of nitrate ions using TiO_2_ nanocrystals embedded in SiO_2_ matrices.^50^ It was found that the urea production was influenced by the kind of solvents, including ethylene glycol monoethyl ether, acetonitrile, sulfolane, and water. From the results, the highest polarity solvent, which was water, yielded the highest amounts of urea. It was explained that these results may be due to the different degrees of dissociation of LiNO_3_ in solvents. With the increase of solvent polarity, the dissociation degree of LiNO_3_ increased, and the amount of NO_3_^−^ available for the reduction reaction on TiO_2_ also increased. Aligned with their previous report, the primary conclusion of this study was that the reduction of NO_3_^−^ was the rate-determining step in the photocatalytic production of urea.

**Fig. 4:**
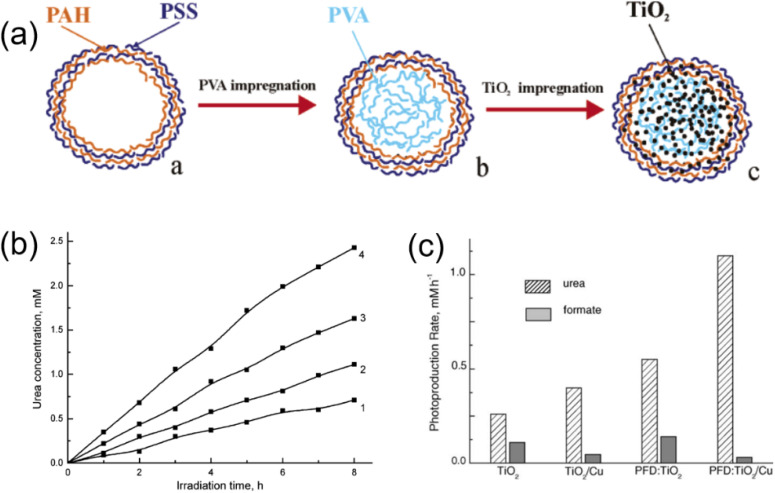
(a) Schematic illustration of the assembly of a photocatalytic microreactor. (b) Time course of the photoproduction of urea in CO_2_-saturated TiO_2_/Cu suspension (1) and inside TiO_2_/Cu-loaded polyelectrolyte capsules of 8.1 (2), 4.2 (3), and 2.2 µm (4) diameter. Reproduced with permission from ref. 51. Copyright 2005, American Chemical Society. (c) Initial rate of urea and formate photoproduction in CO_2_-saturated TiO_2_ suspension, suspension of Cu-modified TiO_2_, PFD-in-water emulsion stabilized with TiO_2_, and PFD-in-water emulsion stabilized with Cu-modified TiO_2_. Aquatic phase initially contained 1 M NaNO_3_ and 1 M isopropanol, pH 5.5. Reproduced with permission from ref. 37. Copyright 2005, Elsevier.

In 2005, the photosynthesis of urea from CO_2_ and NO_3_^−^ was reported by Shchukin *et al.* on TiO_2_/Cu particles encapsulated inside poly(styrene sulfonate)/poly(allylamine hydrochloride) capsules of different size (2.2, 4.2, and 8.1 µm) in aqueous solution.^51^ Poly(vinylalcohol) was employed as electron donor to facilitate the photosynthetic process. Fig. 4a showed an assembly scheme for spherical microreactor. The highest yield of urea photosynthesis (1.7 mM) was achieved for Cu-modified TiO_2_ nanoparticles encapsulated inside 2.2 µm poly(styrene sulfonate)/poly(allylamine hydrochloride) capsules (Fig. 4b). The decreasing the size of the confined microvolume of polyelectrolyte capsules accelerated the NO_3_^−^ photoreduction, which was considered to be a limiting stage of the urea photosynthesis, and correspondingly improving the efficiency of urea production.

The same year, Ustinovich *et al.* reported that urea was synthesized by the photoinduced reduction of CO_2_ in the presence of NO_3_^−^, using titania-stabilized perfluorodecalin-in-water (PFD:TiO_2_) emulsions and isopropanol as the hole scavenging reagent.^52^ The production of urea was obtained by irradiation PFD:TiO_2_ emulsion and TiO_2_ suspension, evidenced that in the case of emulsion the efficiency of urea photoproduction dramatically increased (Fig. 4c). Cu-modified nanodispersed TiO_2_ emulsion also studied the simultaneous photoreduction of CO_2_ and NO_3_^−^, the urea photoproduction rate reached its maximum at copper loading of *ca.*3wt% and was higher than that of PFD:TiO_2_ emulsion (Fig. 4c). The increase of efficiency and selectivity of urea photoproduction observed for titania-stabilized perfluorocarbon-in-water emulsion can be attributed to high concentration of CO_2_ in the oleic phase contacting with photocatalyst and favourable conditions for stabilizing of the reaction intermediates to form C–N bonds in the case of two-phase reaction medium.

**Fig. 5:**
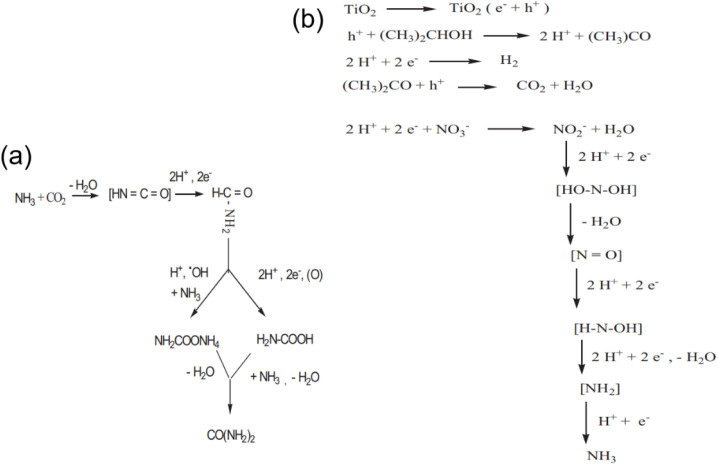
(a) Speculative mechanism for urea formation. (b) Schematic representation and stabilization of intermediate species during nitrate reduction and prevention of ammonia oxidation over TiO_2_-zeolite. Reproduced with permission from ref. 12. Copyright 2011, The American Society of Photobiology.

Despite the inspiring findings, early studies lacked discussion of the reaction mechanism. It was not until 2011 that Srinivas *et al.* first proposed a reaction pathway in his study on the photocatalytic synthesis of urea (Fig. 5a). Firstly, they investigated influence of hole scavengers (isopropanol and oxalic acid) on nitrate reduction for urea formation over TiO_2_ catalyst.^12^ The carbonaceous hole scavengers not only served as electron donors but also underwent further oxidation to produce CO_2_, thus serving as a carbon source, whereas the NO_3_^−^ ion was used as the nitrogen source in their work (Fig. 5b). The results showed that the urea yield slightly increases under the isopropanol as the hole scavenger because the formation of ammonium oxalate, which was not desired under the oxalic acid as the hole scavenger, was avoided. To improve the adsorption of nitrate and to prevent the oxidation of ammonia, which was formed during nitrate reduction, the immobilization of TiO_2_ on zeolite was envisaged. The high activity of the TiO_2_ (10 wt%) supported over zeolite sample can be ascribed to the high dispersion of TiO_2_ on the surface, strong adsorption of substrates and also because of lower recombination of electron–hole pairs generated.

**Fig. 6:**
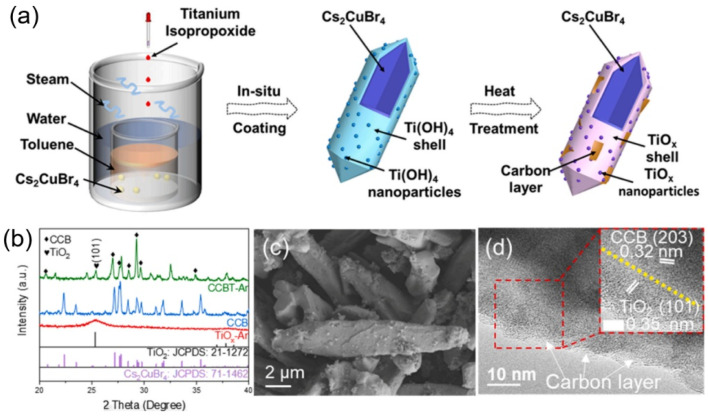
(a) Schematic illustration of the synthesis procedure of CCBT-ar/air catalysts. (b) XRD patterns of CCBT-Ar, CCB, and TiOx-Ar. (c) SEM image of CCBT-Ar. (d) High-resolution TEM image of CCBT-Ar (with a 5 nm bar size inset). Reproduced with permission from ref. 53. Copyright 2025, Elsevier.

Huang *et al.* presented the novel Cs_2_CuBr_4_/TiO_*x*_-Ar (CCBT-Ar) photocatalyst for urea generation through the simultaneous reduction of CO_2_ and NO_3_^−^.^53^ The synthesis of the designed CCBT-Ar catalysts was accomplished through a facile two-step method (as depicted in Fig. 6a). The X-ray diffraction (XRD) patterns showed that CCB was confined with amorphous TiO_*x*_ (Fig. 6b). The scanning electron microscopy (SEM) of the CCBT-Ar adopted a spindle-like shape (Fig. 6c). The research findings demonstrated that the introduction of oxygen vacancies (O_v_) in TiO_*x*_ played a crucial role in reactant adsorption and the promotion of rate-determining steps in urea generation. However, the presence of O_v_ also led to significant carrier trapping effects, reducing the overall reaction activity. The well-designed CCBT-Ar catalyst exhibits outstanding photocatalytic urea generation activity, which attributes to its unique structural synergy. The TiO_*x*_ nanocrystals were interconnected by *in situ* grown carbon nanosheets (Fig. 6d), allowing for efficient electron extraction from TiO_*x*_ and acting as an electron reservoir, effectively suppressing O_v_-induced carrier recombination. As electrons in TiO_*x*_ were consumed, those reserved in the carbon nanosheets were replenished, ensuring the sustainability of the reaction.

**Fig. 7:**
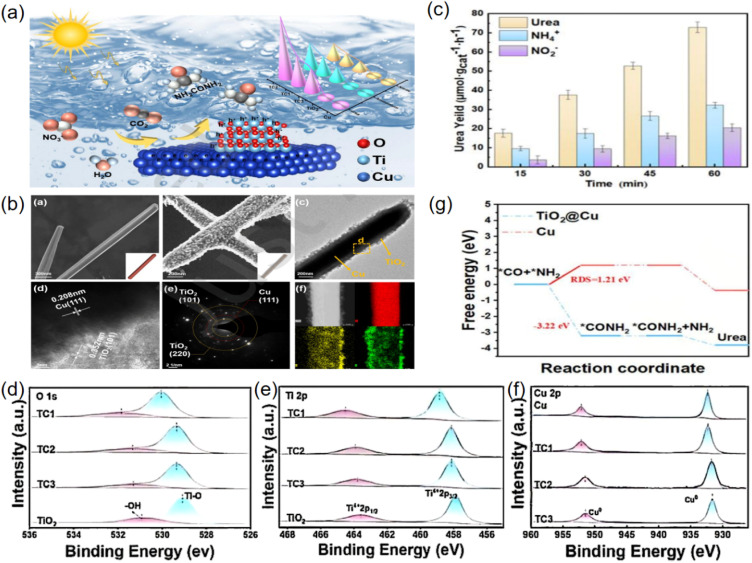
(a) Schematic diagram of photocatalytic co-reduction of nitrate ions and carbon dioxide for urea synthesis over TiO_2_@Cu nanorod. (b) Structure characterization. (SEM image of Cu, the SEM image of TC2, TEM image of TC2, HRTEM image of TC2, SAED image of TC2; the elemental mapping of TC2). (c) The yield of urea over TC2. (d) O 1s (e) Ti 2p and (f) Cu 2p for TC1, TC2, TC3, and TiO_2_ nanoparticle. (g) Free energy diagrams for the electrocatalytic C–N coupling of *CO and *NH_2_ on TiO_2_@Cu and Cu. Reproduced with permission from ref. 54. Copyright 2025, Tsinghua University Press.

Li and coworkers engineered a TiO_2_ nanoparticle modified Cu nanorod photocatalyst (TiO_2_@Cu) for simultaneously promoting the NO_3_^−^ reduction and CO_2_ reduction reaction in the photocatalytic synthesis of urea (Fig. 7a).^54^ The TiO_2_ nanoparticles were uniformly covered onto the surface of the Cu nanorod *via* a impregnation-reduction method (Fig. 7b), and the well-integrated core–shell TiO_2_@Cu showed excellent efficiency in photocatalytic urea synthesis, reaching up to 72.8 µmol g^−1^ h^−1^ of urea yield (Fig. 7c). The remarkable photoactivity was attributed to the unique Ti–O–Cu bond in heterojunction interface of TiO_2_@Cu (Fig. 7d–f), and Ti–O–Cu bond provided a favorable electron transfer pathway from TiO_2_ to Cu, which accelerated the transfer of photogenerated charge and reduced the recombination of hole and electron. Meanwhile, the introduction of Cu altered the energy band structure of TiO_2_, resulting in a smaller band gap and further improving the utilization of light. The density functional theory calculation (Fig. 7g) indicated that the energy barrier of the C–N coupling reaction in Ti–O–Cu site of TiO_2_@Cu (−3.22 eV) was much lower than individual Cu site (1.21 eV).

**Fig. 8:**
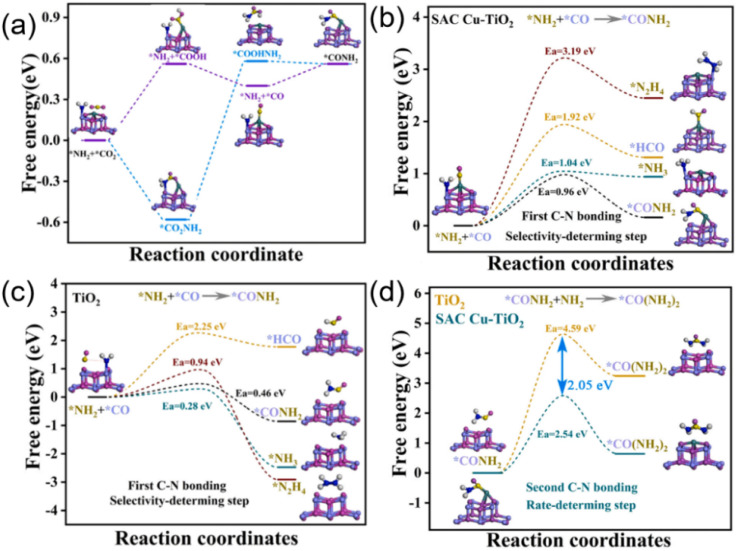
(a) Free energy of formation *CONH_2_ from *NH_2_ and *CO_2_, diagram of free energy changes and activation barriers of *CO and *NH_2_ coupled to CONH_2_ and other parallel CO_2_/NO_3_^−^ reduction reaction on (b) SAC Cu–TiO_2_, (c) TiO_2_, (d) *CO(NH_2_)_2_ formation from CONH_2_ on TiO_2_ and SAC Cu–TiO_2_. Reproduced with permission from ref. 39. Copyright 2023, Elsevier.

It is hard to achieve the simultaneously catalytic reduction of two reactants CO_2_ and nitrate at a single active site. Therefore, constructing bimetallic active sites is a feasible method to simultaneously catalyze the reduction of CO_2_ and nitrate to C–N bonding.^55^ However, due to the long distance between the coupling reaction intermediates generated at the bimetallic sites in the alloy, it is difficult to achieve efficient coupling between CO_2_ and nitrate reduction intermediates. Hence, structuring bimetallic active site with short distance for the simultaneous catalytic co-reduction of CO_2_ and nitrate is conducive to the C–N coupling between reduction intermediate of CO_2_ and nitrate for urea synthesis. Zhao *et al.* engineered dual metal Cu and Ti active sites with a short distance of 2 Å by single atom Cu anchored on TiO_2_ toward photoelectrocatalytic urea synthesis from CO_2_ and nitrate.^39^ Cu and Ti dual active sites can efficiently catalyze the reduction of CO_2_ to *CO and reduction of nitrate to *NH_2_ intermediates, respectively. The relatively short distance of the Ti and Cu double site on SAC Cu–TiO_2_ was conducive to the coupling of the two reaction intermediates *CO and *NH_2_ to formation urea *via* C–N bonding by strong nucleophilic attack of *NH_2_. Density functional theory (DFT) calculations (Fig. 8) verified that compared with parallel competing reactions of *CO and *NH_2_ such as *CO hydrogenation, *NH_2_ hydrogenation and its dimerization, the coupling of *CO and *NH_2_ had a lower energy barrier on dual metal active sites with short distance by single atom Cu anchored on TiO_2_.

**Fig. 9:**
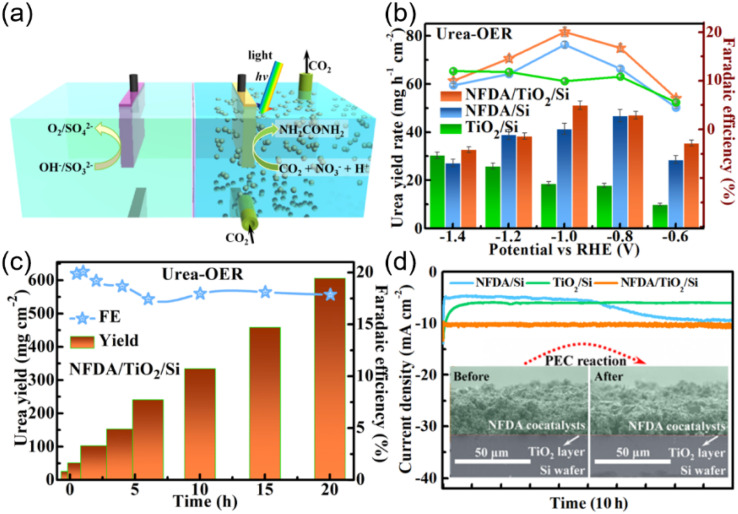
PEC urea synthesis of the Si-based photocathodes in a 0.1 M KHCO_3_^+^ 0.05 M KNO_3_ electrolyte. (a) Diagrammatic sketch of PEC urea synthesis under 1 sun illumination. (b) Urea yield rate (column diagrams) and FE (point plots) of NFDA/TiO_2_/Si (orange), NFDA/Si (blue), and TiO_2_/Si (green) at various potentials for 0.5 h. (c) Time dependence of urea yield (column diagrams) and FE (point plots) acquired from NFDA/TiO_2_/Si held at −1.0 V *vs.* RHE. (d) *J*–*t* curves of NFDA/TiO_2_/Si, NFDA/Si, and TiO_2_/Si at −1.0 V *vs.* RHE for 10 h. The Insets are the cross-sectional FESEM images of NFDA/TiO_2_/Si before and after 10-h PEC reactions. Reproduced with permission from ref. 56. Copyright 2024, PNAS.

**Fig. 10:**
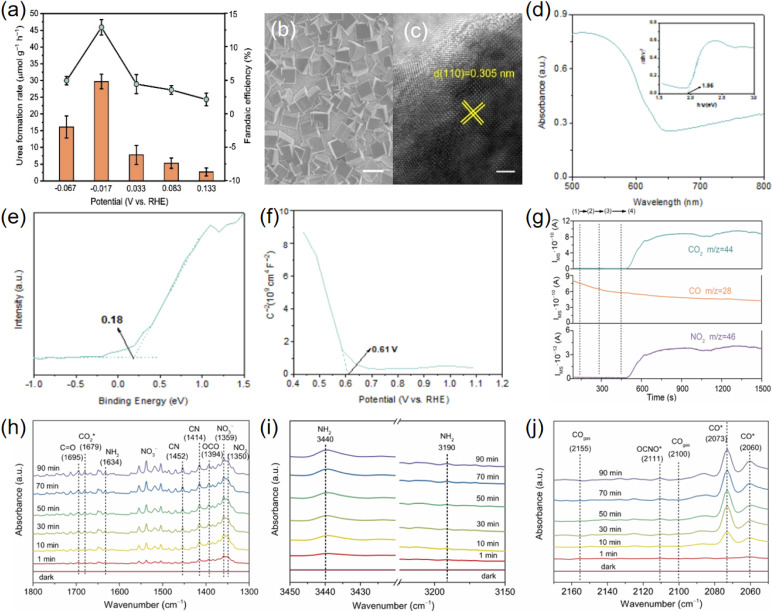
(a) Urea formation rate and FE at different external potentials over Cu_2_O. (b) SEM image of Cu_2_O. (c) TEM image of Cu_2_O. (d) Diffuse reflectance ultraviolet-visible spectrum and the corresponding (αhν)^2^*versus* photon energy plot of Cu_2_O. (e) VB XPS spectrum of Cu_2_O. (f) Mott–Schottky plots of Cu_2_O. (g) Photo-assisted DEMS measurements over Cu_2_O. Reaction conditions: (1) without external bias and CO_2_ in darkness; (2) with cathodic bias but without CO_2_ in darkness; (3) with cathodic bias but without CO_2_ under light irradiation (AM 1.5 G irradiation); (4) with cathodic bias and CO_2_ under light irradiation. *In situ* FTIR spectra in the range of (h) 1300–1800 cm^−1^, (i) 3150–3450 cm^−1^ and (j) 2050–2170 cm^−1^. Reproduced with permission from ref. 6. Copyright 2024, Wiley-VCH.

The photoelectrochemical (PEC) system can be a viable solution for green synthesis of urea by combining the light absorbers and the catalysts into a fully integrated electrode. Thus, exploring and developing the PEC device is significant for green and efficient synthesis of urea in aqueous solution under mild conditions *via* solar energy. Wang *et al.* designed a hierarchical-structured Si-based photocathode to efficiently drive the solar-to-urea conversion by coupling the NO_3_^−^and CO_2_, in which nanostructured n^+^p-Si serves as the light absorber decorated with TiO_2_ layer and NiFe diatomic cocatalysts on N-doped carbon nanosheets (Fig. 9a).^56^ The results demonstrated a remarkably high urea yield rate, faradaic efficiency (FE), and stable operation time of 81.1 mg h^−1^ cm^−2^, 24.2%, and 20 h at −1.0 V *vs.* RHE, respectively (Fig. 9b–d). The synergetic effect of NFDA, TiO_2_ layer, and n^+^p-Si enhances the charge-carrier dynamics of the photocathode. Duan *et al.* reported a photoelectrochemical method for urea synthesis by co-reduction of carbon dioxide and nitrate ion over a Cu_2_O photocathode,^6^ delivering urea formation rate of 29.71 ± 2.20 µmol g^−1^ h^−1^ and faradaic efficiency (FE) of 12.90 ± 1.15% at low external potential (0.017 V *vs.* Reversible hydrogen electrode) (Fig. 10a). The Cu_2_O exhibited cubic morphology and exposes (100) facet (Fig. 10b and c), with suitable conduction band (CB) position (at about 1.1 V *vs.* SHE) to co-reduce CO_2_ and NO_3_^−^ (Fig. 10d–f). *In situ* Fourier transform infrared spectroscopy (Fig. 10h–j) and photo-assisted online differential electrochemical mass spectrometry (Fig. 10g) showed that CO* and 
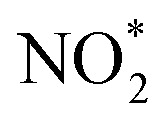
 species were the key intermediates for the subsequent C–N coupling. Density functional theory (DFT) calculations revealed that the first CN coupling during urea synthesis took places between CO* and 
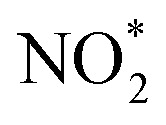
, which were the rate determining step, and the second C–N coupling occurred between CONH* and 
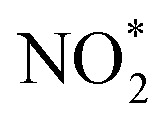
. Zhao demonstrated an efficient GaN/Si photoelectrode for PEC urea synthesis from simultaneous NO_3_^−^ and CO_2_ reduction reactions under solar light.^57^ The built-inpotential in n^+^−p Si and the inherent catalytic activity of GaN nanowires (NWs) led to the selective synthesis of urea at a low overpotential.

### 3.2. Urea synthesis from CO_2_ and N_2_

The conversion of N_2_ and CO_2_ into urea through a photocatalytic C–N coupling reaction under ambient conditions presents a favorable approach. On the one hand, N_2_ can serve as an abundant nitrogen source for urea photosynthesis, which accounts for 78% of the atmosphere. On the other hand, CO_2_, a main greenhouse gas leading to serious environmental concerns, can serve as a carbon source. Thus, the approach not only achieves effective energy conservation but also mitigates environmental concerns. Nevertheless, the photocatalytic urea production process still suffers from extremely serious challenges. Rationally designing photocatalysts that integrate the capture of inert gas molecules, bond cleavage and C–N coupling capabilities is a crucial prerequisite to promote the performance of urea photosynthesis.

**Fig. 11:**
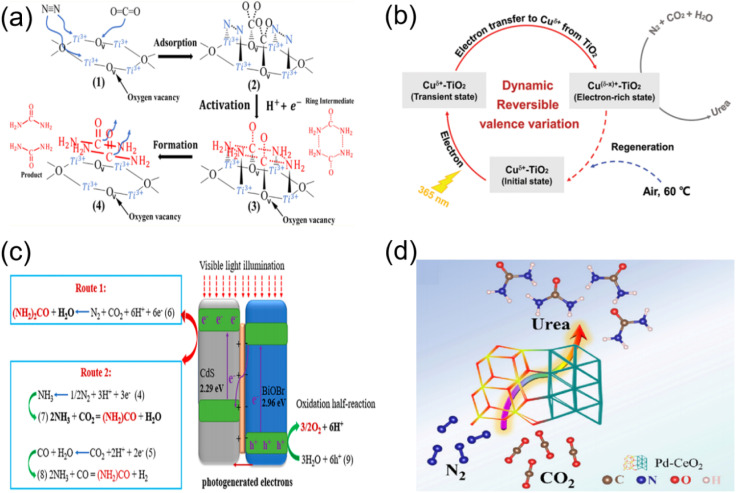
(a) Mechanism of photocatalytic coreduction of N_2_/CO_2_ to form CO(NH_2_)_2_. Reproduced with permission from ref. 58. Copyright 2021, American Chemical Society. (b) Proposed mechanism of reversible and cooperative photocatalysis in Cu SA-TiO_2_. Reproduced with permission from ref. 43. Copyright 2022, Wiley-VCH. (c) Suggested mechanism for the photocatalytic synthesis of urea on 40% 2D-CdS@3D-BiOBr. Reproduced with permission from ref. 59. Copyright 2023, American Chemical Society. (d) Schematic diagram of the photocatalytic C–N coupling reaction in urea synthesis through the conversion of N_2_ and CO_2_. Reproduced with permission from ref. 44. Copyright 2023, The Royal Society of Chemistry.

In 2021, Maimaiti successfully established the photocatalytic synthesis of urea for the first time in the N_2_/CO_2_ system.^58^ They utilized oxygen vacancy-rich TiO_2_ loaded on carbon nanotubes with Fe cores (Ti^3+^–TiO_2_/Fe-CNTs) as the catalyst and achieved the coreduction of N_2_ and CO_2_ into urea in water without the addition of a hole scavenger. The authors identified Ti^3+^ sites and the adjacent oxygen vacancy serves as the active center for N_2_ and CO_2_, respectively. Adsorbed N_2_ and CO_2_ are further activated by photogenerated electrons, forming six-membered cyclic intermediates (H_2_NCONH_2_)_2_, which eventually evolved into urea (Fig. 11a).

It can be concluded from photocatalytic reactions involving multiple electrons (such as N_2_ fixation and CO_2_ reduction) that the efficient extraction of photogenerated electrons is an essential prerequisite for advanced photocatalysis. However, low availability of photogenerated electrons in intrinsic photocatalysts seriously hinders its further application. Zhang *et al.* reported a photoinduced strategy based on a TiO_2_ photocatalyst-immobilized reversible single-atom copper (denoted as Cu SA-TiO_2_) for the photocatalytic synthesis of urea using N_2_ and CO_2_ molecules in the presence of pure H2O (Fig. 11b).^43^ The introduction of reversible single-atom Cu in the designed sample imparted additional electron-rich sites for the photoactivation of reactants (N_2_, CO_2_, and H_2_O) and C–N coupling, together with the accelerated electron-transfer dynamics, ensuring the multi-electron supply for urea photosynthesis from N_2_, CO_2_, and H_2_O, thereby promoting urea photosynthesis.

Theoretically, integrating N_2_ reduction reaction semiconductors and CO_2_ reduction reaction semiconductors to construct composite photocatalytic materials is expected to enable the conversion of CO_2_ and N_2_ into urea under photocatalytic conditions. For example, Wang *et al.* prepared 2D-CdS@3D-BiOBr S-scheme heterostructures by self-assembly of BiOBr microspheres (3D-BiOBr) and CdS nanosheets (2D-CdS). It was proposed that Cd^2+^ in CdS and oxygen vacancies in BiOBr of 2D-CdS@3D-BiOBr hybrids facilitate the adsorption and activation of N_2_ and CO_2_, respectively, resulting in the formation of the *HNCONH intermediate.^59^ Subsequently, their group synthesized the CdS@Bi_2_WO_6_ heterojunction for photocatalytic CO_2_–N_2_–H_2_O to urea with visible light. It was suggested that urea was synthesized on the surface of CdS through two mechanisms: (I) the reaction between the activated intermediates of CO_2_ and N_2_ (mainly) and (II) the reaction of the *in situ* formed NH_3_ with the feed CO_2_ (Fig. 11c).

**Fig. 12:**
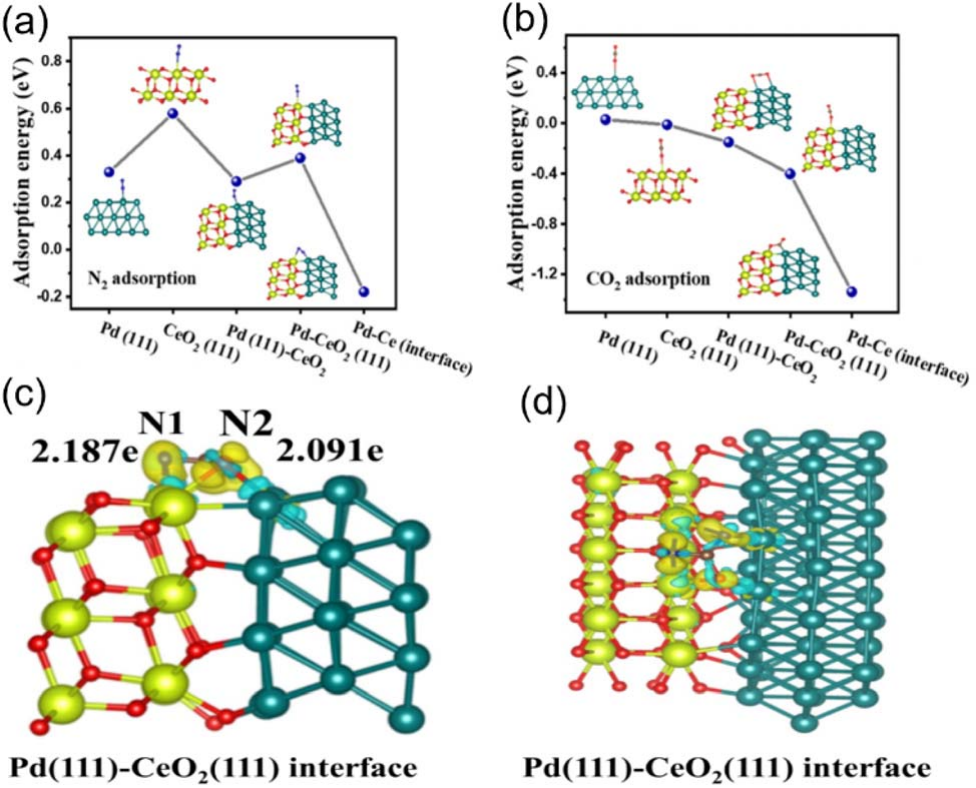
(a) The adsorption energy of N_2_ at different reaction sites. (b) The adsorption energy of CO_2_ at different reaction sites. The differential charge density of *NCON at Pd(111)-CeO_2_ (111) interface. (c) Side view, (d) top view. (The blue color represents electron consumption and the yellow color represents electron accumulation). Reproduced with permission from ref. 44. Copyright 2023, The Royal Society of Chemistry.

**Fig. 13:**
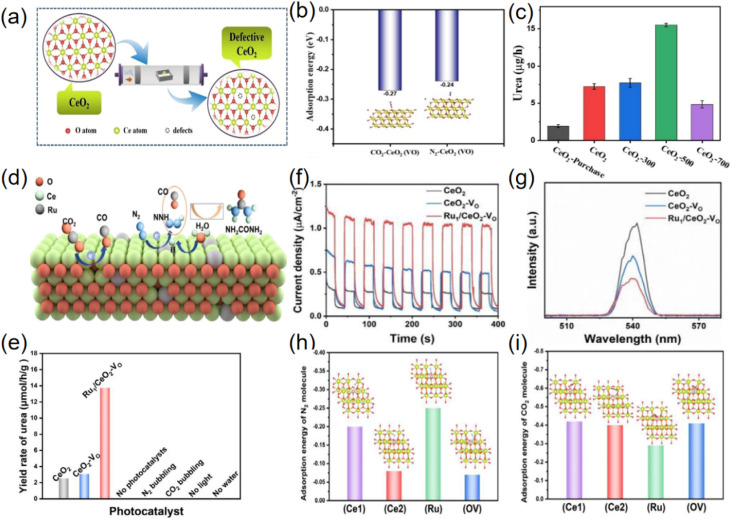
(a) Schematic diagram of oxygen vacancy generation. (b) Adsorption energy of N_2_ and CO_2_ on CeO_2_ with oxygen vacancy. (c) The urea formation rates of CeO_2_-purchase (commercial), CeO_2_, CeO_2_-300, CeO_2_-500 and CeO_2_-700 with all optical spectrum as light source. Reproduced with permission from ref. 63. Copyright 2023, Wiley-VCH. (d) Mechanism of the photocatalytic urea synthesis based on synergistic effects of the Ru1/CeO_2_-VO. (e) The prepared materials and urea production efficiency under different conditions. (f) Photocurrent responses of CeO_2_, CeO_2_–VO, and Ru_1_/CeO_2_–VO. (g) PL spectra of CeO_2_, CeO_2_–VO, and Ru_1_/CeO_2_–VO. (h) N_2_ adsorption energy of different sites. (i) CO_2_ adsorption energy of different sites. Reproduced with permission from ref. 64. Copyright 2025, Wiley-VCH.

The weak adsorption and activation ability of inert gases (CO_2_ and N_2_) on photocatalysts has been the main challenge hindering the development of this technology.^60–62^ Niu designed a Pd-decorated CeO_2_ photocatalyst for the photoinduced coreduction of N_2_ and CO_2_ into urea,^44^ enabling spontaneous electron transfer at the palladium–ceria interface (Fig. 11d). The investigations further endorsed that the emerged space-charge region in the CeO_2_(111)/Pd(111) interface not only effectively facilitates the targeted capture and activation of inert CO_2_ and N_2_ but also stabilizes the formation of key intermediates (*NCON) (Fig. 12). Subsequently, they established mesoporous CeO_2−*x*_ nanorods with adjustable oxygen vacancy concentration by heat treatment in Ar/H_2_ (90%:10%) atmosphere (Fig. 13a) and served as photocatalysts to convert both CO_2_ and N_2_ into urea under ambient conditions.^63^ By introducing oxygen vacancies to enhance the targeted adsorption and activation of N_2_ and CO_2_ (Fig. 13b), CeO_2_-500 (CeO_2_ nanorods heated treatment at 500 °C) revealed high photocatalytic activity toward the C–N coupling reaction for urea synthesis with a remarkable urea yield rate of 15.5 µg h^−1^ (Fig. 13c). In the most recent study, Meng synthesized a single-atom Ru and oxygen vacancies co-modified CeO_2_ (Ru_1_/CeO_2_–VO) by a photochemical strategy to achieve photocatalytic simultaneous reduction of CO_2_ and N_2_ for the synthesis of urea (Fig. 13d).^64^ Benefiting from the synergistic effect of Ce^3+^–VO and single atoms, Ru_1_/CeO_2_–VO nanosheets delivered the highest urea yield rate of 13.73 µmol g^−1^ h^−1^ (Fig. 13e). Strong electron metal support interactions between the Ru single atoms and CeO_2_ enabled effective separation of photoexcited carriers (Fig. 13f and g). Importantly, it was confirmed by experimental characterization and DFT calculations (Fig. 13h and i) that the vacancies can effectively adsorb CO_2_, and Ru single atoms can promote N_2_ adsorption and activation and contribute to hydrolysis dissociation, supplying protons for hydrogenation of active species.

**Fig. 14:**
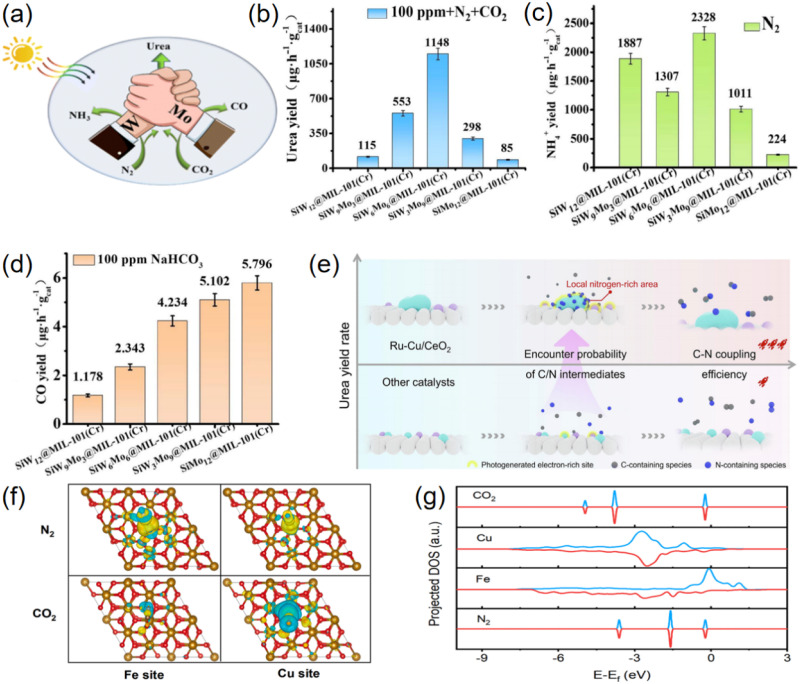
(a) Schematic diagram of the design strategy for dual-active site catalysts in urea synthesis; (b) the urea production rate of composites in 100 ppm NaHCO_3_; (c) the NH_4_^+^ production rate of the composites in N_2_; (d) the CO production rates of the composites in 100 ppm NaHCO_3_. Reproduced with permission from ref. 72. Copyright 2024, The Royal Society of Chemistry. (e) The comparison between the designed Ru–Cu/CeO_2_ catalysts (above) and the reported multi-site catalysts (below) during urea synthesis. Reproduced with permission from ref. 73. Copyright 2025, Elsevier. (f) Difference charge-density stereograms of adsorbed N_2_ and CO_2_ on the Cu and Fe sites, respectively. Yellow and cyan represent electron accumulation and depletion, respectively. (g) Calculated projected density of states of the d-orbital of Cu and Fe in CuL-Fe_2_O_3_, and CO_2_/N_2_ molecular orbitals. Reproduced with permission from ref. 37. Copyright 2025, Elsevier.

The use of metal elements as single active sites for the reduction of N_2_ or CO_2_ has been extensively studied and reported.^65–68^ However, urea synthesis requires the coupling reaction of the key intermediates *CO and *N to produce C–N bonds. Due to their high reactivity, these crucial reaction intermediates cannot exist in a stable form within the reaction system.^42,69–71^ Therefore, maintaining an optimal distance between the two reaction sites to enable the interaction of intermediates is an excellent strategy. Luo proposed a catalyst design strategy with dual active sites to meet the needs of urea synthesis (Fig. 14a). A series of composites (SiW_12−*X*_Mo_*X*_@MIL101(Cr), *X* = 0, 3, 6, 9, 12) were obtained and applied for photocatalytic urea synthesis from N_2_ and CO2.^72^ The urea production rate of SiW_6_Mo_6_@MIL-101(Cr) reaches 1148 µg h^−1^ g_cat_^−1^ under the optimal experimental conditions (Fig. 14b). The performance from both experimental (Fig. 14c and d) and DFT calculation results (Table 1) indicated that the W site in SiW_6_Mo_6_ was assigned for the activation of nitrogen, whereas the Mo site was assigned for the activation of CO_2_. Zheng developed a multi-site photocatalyst, consisting of CeO_2_ nanorods decorated with Ru nanoparticles and Cu single atoms (Ru–Cu/CeO_2_), for the purpose of synthesizing urea at high yield.^73^ The incorporation of Ru and Cu sites was crucial not only to generate high-density photogenerated electrons, but also to facilitated N_2_ and CO_2_ adsorption and conversion. The *in situ* formed local nitrogen-rich area at Ru sites increased the encounter possibility with the carbon-containing species generated from Cu sites, substantially promoting C–N coupling (Fig. 14e). Lu reported a Cu single-atom-decorated porous Fe_2_O_3_ nanorod catalyst with a Cu–O–Fe configuration for the direct artificial photocatalytic synthesis of urea from N_2_ and CO_2_ in pure water.^37^ Because the d-orbitals of the Cu/Fe sites were close to the molecular orbitals of CO_2_/N_2_, the CuFe dual active sites can selectively adsorb and activate N_2_/CO_2_, thereby facilitating efficient C–N coupling (Fig. 14f and g).

**Table 1:** Preparation of the catalysts under different conditions. Reproduced with permission from ref. 72. Copyright 2024, The Royal Society of Chemistry

Species	*E*_total_ (ev)	*E*_mol−gas_ (ev)	*E*_sub-SiW6Mo6_ (ev)	*E*_ads_ (ev)
Mo–CO_2_	471.519	−22.958	−448.034	−0.527
W–CO_2_	−471.303	−22.958	−448.034	−0.311
Mo–N_2_	−465.620	−17.096	−448.034	−0.490
W–N_2_	−465.968	−17.096	−448.034	−0.838

**Fig. 15:**
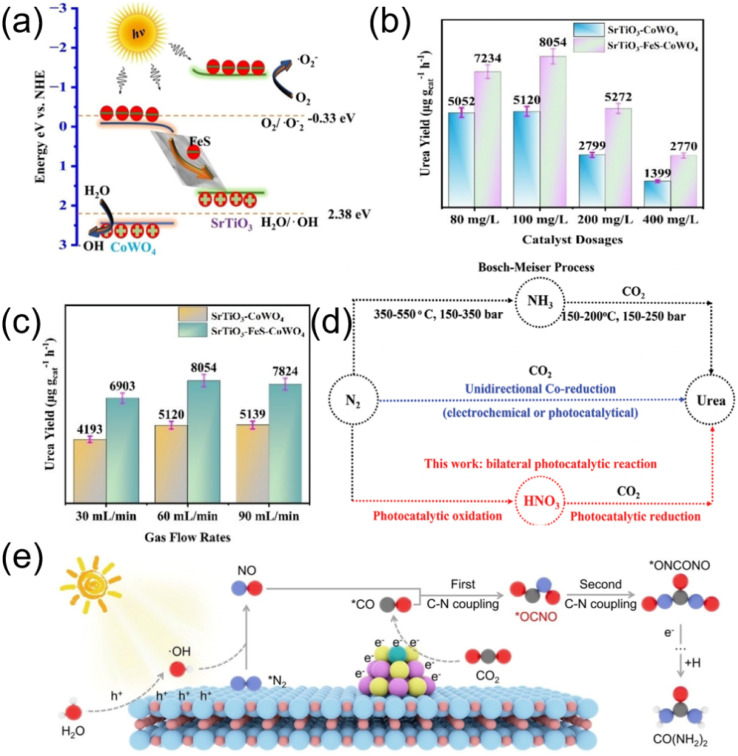
(a) Energy band diagram of SrTiO_3_–FeS–CoWO_4_*Z*-scheme heterojunctions. (b) Urea yields of SrTiO_3_–FeS–CoWO_4_ with different catalyst dosages; (c) urea yields of SrTiO_3_–FeS–CoWO_4_ with different gas-flow rates. Reproduced with permission from ref. 82. Copyright 2024, Wiley-VCH. (d) Comparison of the reaction conditions of different routes for urea synthesis. Reproduced with permission from ref. 48. Copyright 2024, Wiley-VCH. (e) Schematic illustration of photocatalytic urea synthesis mechanism over Ni_1_–CdS/WO_3_ catalyst. Reproduced with permission from ref. 45. Copyright 2024, Wiley-VCH.

Photocatalysts with high reduction activity are necessary for overcoming the unfavorable energy barrier.^42,74^ It is well known that the *Z*-scheme photocatalyst can retain the high reduction capability of materials in the heterojunction.^75–78^ The high reduction activity favors boosting kinetics for co-reduction of N_2_ and CO_2_ and promoting C–N coupling reaction. Meanwhile, the *Z*-scheme photocatalyst has good charge separation ability, which can inhibit charge recombination and accelerate electron transfer.^79–81^ In this regard, the *Z*-scheme photocatalyst could be more reasonable for improving photocatalytic urea synthesis by co-reducing CO_2_ and N_2_. SrTiO_3_–FeS–CoWO_4_*Z*-scheme photocatalyst was designed to promote urea synthesis by N_2_ and CO_2_ co-reduction in water (Fig. 15a).^82^ Its fast electron transfer overcomes the bottleneck of sluggish kinetics for C–N coupling reaction, and dual active sites for the adsorption and activation of N_2_ and CO_2_ enhanced the kinetics for urea synthesis, resulting in enhanced urea yield of 8054.2 µg g_cat_^−1^·h^−1^ on SrTiO_3_–FeS–CoWO_4_ (Fig. 15b and c).

The disparity in physical properties, structure, and catalytic kinetics between N_2_ and CO_2_ poses stringent demands for the design of key catalysts, reaction control, mass transfer, and other aspects. Considering the unique advantages of nitrogen oxidation activation and the bilateral redox of photocatalytic reactions, Ding *et al.* synthesized a Ru–TiO_2_ photocatalyst for urea photo-synthesis through a nitric acid-mediated pathway combining nitrogen oxidation and subsequent kinetically advantageous nitrate and CO_2_ co-reduction (Fig. 15d).^48^ The remarkable photo-activity was attributed to its unique oxygen vacancy-anchored Ru nanostructure (Ru–O_4_Ti_1_), which effectively activates inert N_2_ molecules to minish the disparity of orbital energy levels, facilitated the formation of crucial *NN(OH) intermediates, and served as an “electronic pump” to avoid electronegativity effect for facilitating electron transfer from nitrogen to TiO_2_ support for urea photosynthesis.

Typically, the urea synthesis from N_2_ and CO_2_ is regarded as a co-reduction process. Current works mainly focused on the photogenerated electrons, the role of photogenerated holes has been commonly ignored. If photogenerated holes could be employed in activating reactant, it would not only mitigate the competition between N_2_ and CO_2_ in photogenerated electrons, but also improve the utilization efficiency of photogenerated carriers toward urea product, thereby boosting the performance of photocatalytic urea synthesis. Zheng *et al.* constructed a redox heterojunction consisting of WO_3_ and Ni single-atom decorated CdS (Ni_1_–CdS/WO_3_),^45^ enabling photogenerated electrons and holes to participate in the conversion of CO_2_ and N_2_ respectively (Fig. 15e). Specifically, the N_2_ was activated by photogenerated holes and CO_2_ was converted by photogenerated electrons during photocatalytic urea synthesis, which mitigated the competition of photogenerated electrons between N_2_ and CO_2_, enhanced the utilization efficiency of photogenerated carriers for urea products, and ultimately promoted the synthesis of urea.

### 3.3. Urea synthesis from CO_2_ and NH_3_

**Fig. 16:**
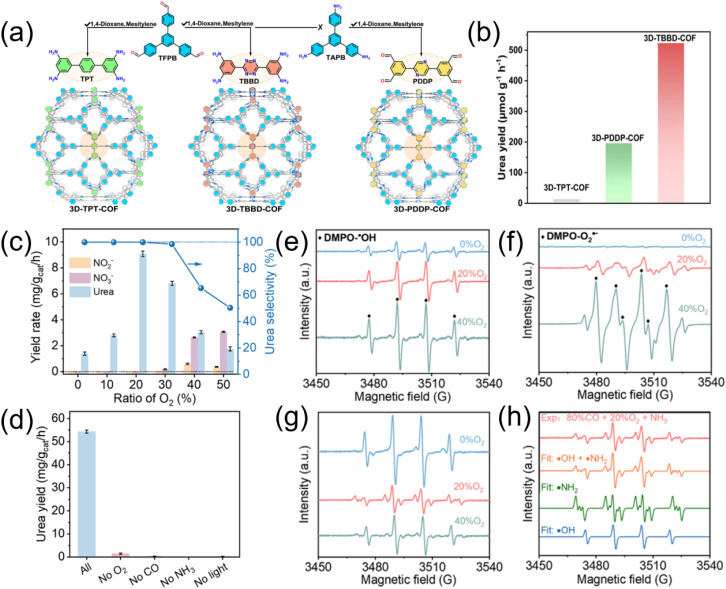
(a) Schematic synthesis of 3D-TPT-COF (green), 3D-TBBD-COF (red) and 3D-PDDP-COF (yellow); (b) urea formation rate using 3DTPT-COF, 3D-PDDP-COF, and 3D-TBBD-COF as photocatalyst. Reproduced with permission from ref. 38. Copyright 2025, Nature. (c) Dependence of urea yield and selectivity on the ratio of O_2_ in the atmosphere; EPR measurements of reactive radicals generated during photocatalysis on P25-4 h using DMPO as the trapping agent: (d) detection of ˙OH in pure aqueous solution under different atmospheric O_2_ ratios; (e) detection of O_2_˙^−^ in methanol solution under different atmospheric O_2_ ratios; (f) detection of both ˙OH and ˙NH_2_ in 2 M ammonia aqueous solution under different atmospheric O_2_ ratios. (g) Comparisons of EPR signal obtained in 2 M ammonia under 20% O_2_ (red trace in (f)) with theoretically fitted EPR signals. (h) Performance comparison under the most optimized conditions (20% O_2_, 80% CO and pH = 9) and control systems lacking O_2_, CO, ammonia or light. Reproduced with permission from ref. 46. Copyright 2025, Wiley-VCH.

Compared with the stable N≡N bond in N_2_, the lone pair electrons in NH_3_ are naturally reactive centers, in favor of fast reaction dynamics.^18,83^ As a total result, photosynthesis of urea from NH_3_ and CO_2_ appears more promising for future practical industrial applications.^83–87^ Jiang *et al.* designed and synthesized a series of 3D N-heterocyclic covalent organic frameworks for urea photosynthesis from NH_3_ and CO_2_.^38^ Three isomorphic three-dimensional (3D) COFs with two fold interpenetrated ffc topology (namely3D-TPT-COF, 3D-PDDP-COF, and 3D-TBBD-COF) were functionalized by benzene, pyrazine, and tetrazine active cores, respectively, to modulate the catalytic micro-environment through the change in the number of heterocyclic N atoms on the active cores (Fig. 16a). The geometrical and electronic structural advantages endowed 3D-TBBD-COF with superior photocatalytic activity towards urea production with the yield of 523 µmol g^−1^ h^−1^, 40 and 4 times higher than that for 3D-TPT-COF and 3D-PDDP-COF, respectively (Fig. 16b), indicating the heterocyclic N microenvironment dependent catalytic performance for these COFs photocatalysts.

Besides, Sheng *et al.* presented a photocatalytic pathway for the selective urea synthesis through the oxidative coupling between CO and NH_3_.^46^ They uncovered that the O_2_ concentration played a crucial role in controlling both the urea production rate (Fig. 16c) and its selectivity by effectively controlling the generation of oxidative species (Fig. 16d–g), such as photogenerated holes (h+), superoxide radicals (O_2_˙^−^) and hydroxyl radicals (˙OH). Using oxygen-deficient TiO_2_ under an air-level (20%) O_2_ atmosphere, a urea generation rate of 54.31 mg g_cat_^−1^ h^−1^ with 100% selectivity can be achieved (Fig. 16h). Mechanistic studies reveal that the process began with the oxidation of NH_3_ to ˙NH_2_ through oxidative radicals generated on TiO_2_, especially the oxygen-derived O_2_˙^−^. This ˙NH_2_ radicals then coupled with CO to form urea.

### 3.4. Urea synthesis from other raw materials

The clean-energy-driven synthesis of urea from carbon- and nitrogen-containing small molecules has garnered significant interest but remained great challenges to achieve with high selectivity.

**Fig. 17:**
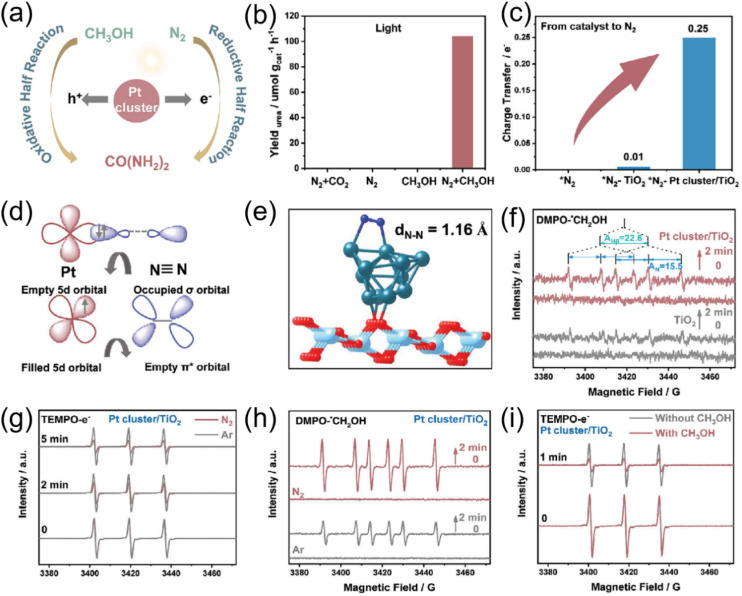
(a) Schematic diagram of the photocatalytic synergistic redox urea synthesis system; (b) blank control experiments of N_2_, CH_3_OH, and light for urea synthesis; (c) bader calculation of N_2_ on TiO_2_ and Pt cluster/TiO_2_; (d) a simplified schematic diagram of N_2_ bonding to a Pt center; (e) schematic diagram of N_2_ on the Pt cluster/TiO_2_ surface; (f) EPR results of the captured oxidative DMPO-CH_2_OH on the TiO_2_ and Pt cluster/TiO_2_; (g) EPR results of reductive TEMPO-e^−^ generation on the Pt cluster/TiO_2_ under N_2_ or Ar atmosphere; (h) EPR results of the captured oxidative DMPO-CH_2_OH on the Pt cluster/TiO_2_ under N_2_ and Ar atmosphere; (i) EPR results of reductive TEMPO-e^−^ generation on the Pt cluster/TiO_2_ with or without CH_3_OH during the photoredox reaction. Reproduced with permission from ref. 40. Copyright 2024, Wiley-VCH.

A Pt cluster-modified TiO_2_ (Pt cluster/TiO_2_) catalyst was designed through the impregnation reduction method to facilitate the photocatalytic synthesis of urea by promoting the simultaneous N_2_ reduction and CH_3_OH oxidation reactions (Fig. 17a).^40^ The prepared Pt cluster/TiO_2_ catalyst exhibited outstanding efficiency in urea synthesis, achieving a rate of 105.68 µmol g^−1^ h^−1^ with N selectivity of 97.29 ± 0.79% (Fig. 17b). Further analysis with density functional theory (DFT) calculation revealed that the “σ–π*” donor–acceptor interaction occurred between Pt clusters and N_2_ (Fig. 17c–e), efficiently reducing the N_2_ hydrogenation barrier. EPR experiments demonstrate that photogenerated electrons (e^−^) and hole (h^+^) were synchronously consumed through N_2_ reduction and CH_3_OH oxidation (Fig. 17f–i), thereby accelerating urea synthesis. The crucial step of C–N coupling was initiated by the reaction between *NH–NH and *CHO intermediate, facilitated by the low energy barrier on Pt cluster/TiO_2_. For comparative analysis, the key performance parameters of photocatalysts employed in urea synthesis, including such metrics as urea yield, stability, and reaction conditions, are summarized in Table 2.

**Table 2:** Comparison of the urea production performance of reported photocatalysts. Reproduced with permission from ref. 23. Copyright 2025, Wiley-VCH

Photocatalysts	Reactants	Urea formation rate [µmol g^−1^ h^−1^]	Light source	AQY	Cyclic stability
Cu SA-TiO_2_	N_2_, CO_2_, H_2_O	7.2	365 nm	—	10 h
Ni_1_–CdS/WO_3_	N_2_, CO_2_	7.8	385 nm	0.15%	10 h
2D-CdS@3D-BiOBr	N_2_, CO_2_, H_2_O	15	Visible light	3.93%	2 h
TiO_2_/10 wt%–Fe_2_TiO_5_	NH_3_, CO_2_	3	UV	—	12 h
SrTiO_3_–FeS–CoWO_4_	N_2_, CO_2_	134.24	UV-vis	—	4 h
Pt cluster/TiO_2_	N_2_, CH_3_OH	105.68	—	—	9 h
Ru–TiO_2_	N_2_, CO_2_	24.95	380 nm	4.7%	3.75 h
Pd–CeO_2_	N_2_, CO_2_	9.2	UV-vis	—	15 h
CeO_2−*x*_	N_2_, CO_2_	15.5	200–400 nm	3.93%	15 h
15%NiCoP–ZnIn_2_S_4−*x*_	N_2_, CO_2_, H_2_O	19.6	420 nm	9.2%	9 h
Ti^3+^–TiO_2_	N_2_, CO_2_, H_2_O	177.53	UV-vis	—	12 h

## 4 Conclusions

Photocatalytic synthesis of urea *via* converting CO_2_ and N_2_/NH_3_/NO_3_^−^ into high-value urea chemical under ambient conditions is a green and sustainable technology driven by clean and renewable energy. In this review, we systematically describe the basic principle details for photocatalytic urea synthesis, including fundamental mechanisms (light absorption by photocatalyst, separation and migration of the photogenerated charge carriers, surface redox reactions-activation of carbon and nitrogen reactants and C–N coupling), identification of key intermediates and product identification and quantification. We emphatically review experimental study, catalyst design and theoretical research progress on urea photosynthesis. Various systems, including CO_2_–N_2_, CO_2_–NO_3_^−^ and CO_2_–NH_3_ systems, *etc.*, have been systematically summarized. Urea has been successfully produced on photocatalyst-like metal oxides (TiO_2_, CeO_2_), single-atom (Cu SA-TiO_2_), MOF (Ce-BTC), inorganic composites (Ti^3+^–TiO_2_/Fe-CNTs) from the conversion of CO_2_ and types of nitrogen sources. To enhance the selectivity and efficiency of photosynthesis of urea, various photocatalysts have been designed and developed, including single-atom catalysts, bimetallic site catalysts, heterojunction catalysts and defect engineering, *etc.* The C–N coupling is a key step in urea synthesis, involving the activation and coupling of CO_2_ and nitrogen sources. The variation of catalysts and reaction conditions can lead to different reaction mechanisms. For instance, direct C–N coupling, where CO_2_ and N_2_ are directly coupled on the catalyst surface to form urea. In addition, indirect C–N coupling means that the nitrogen source is first reduced to intermediates (such as *NH_2_, *NO, *etc.*), and then coupled with *CO produced by the reduction of CO_2_ to form urea. In some cases, the photogenerated electrons and holes respectively activate CO_2_ and N_2_, promoting C–N coupling. Despite the diversity in raw materials, high-performance photocatalysts universally rely on a synergistic combination of three core aspects: (i) optimal adsorption and activation: the catalyst must possess active sites that can effectively adsorb and activate both N_2_ (or other N-sources) and CO_2_ (or other C-sources), often requiring dual or multiple active sites. (ii) Efficient charge separation and migration: a well-designed heterostructure or energy band alignment is crucial to spatially separate photogenerated electrons and holes and direct them to the respective reduction and oxidation sites. (iii) Facilitated C–N coupling: the local microenvironment and electronic structure of the active sites should be tuned to lower the energy barrier for the key C–N coupling step, which is often the rate-determining step. Although some exciting progress has been made, research in this area is still at the infant stage and requires further works. Firstly, the efficiency of C–N coupling is beyond satisfactory due to the difficulty in achieving the co-adsorption, activation and coupling processes of multiple reactants, such as the large difference in activation energy between CO_2_ and N_2_/NH_3_/NO_*x*_. Additionally, there is still controversy regarding the reaction mechanism, particularly concerning the activation of reactants, the formation of key intermediates, and the reaction pathway of the coupling step. Besides, the currently developed photocatalysts for urea photosynthesis mainly response to ultraviolet region. The inherently contradiction between more visible light absorption and the thermodynamic feasibility of urea photosynthesis is largely challenge due to a relatively high potential of the CO_2_RR to form *CO. An ideal future photocatalyst for urea synthesis should embody the following key properties: (i) broad-spectrum solar energy harvesting: the ability to utilize a wider range of the solar spectrum, including visible and even near-infrared light. (ii) Atomic-level precision in active sites: catalysts with well-defined, single-atom or cluster sites tailored for specific reactant adsorption and C–N coupling. (iii) Unprecedented selectivity: near 100% selectivity towards urea, effectively suppressing competing reactions (*e.g.*, NH_3_ emission, H_2_ evolution). (iv) Exceptional long-term stability: robustness against photocorrosion, poisoning, and structural degradation over prolonged operation. Therefore, the design of multifunctional visible response photocatalysts, the development and application of advanced characterization techniques, and the exploration of novel reaction pathways require further research to address these challenges.

In summary, we hope that this review provides an overview of the current status and inspires greater interest in the development of alternative photocatalytic urea synthesis. Meanwhile, it is expected that through continuous innovation in photocatalyst design, optimization of reaction conditions, and in-depth understanding of reaction mechanisms, efficient photocatalytic urea synthesis can be achieved, making contributions to addressing energy crises and environmental issues.

## Author contributions

Miss Peixia Li: writing original draft and conceptualization. Dr Zhidong Yang: supervision and writing review & editing.

## Conflicts of interest

There are no conflicts of interest to declare.

## Data Availability

No primary research results, software or code have been included and no new data were generated or analysed as part of this review.
